# hMSCs-derived exosomal MIR17HG promotes follicular helper T cell differentiation and osteosarcoma progression via the miR-372-3p/BCL6 axis

**DOI:** 10.1016/j.jbo.2025.100726

**Published:** 2025-11-12

**Authors:** Jin Qi, Gang Xue, Baomin Wu, Peng Zhu, Yapeng Wang

**Affiliations:** a Department of Orthopedics, The First Affiliated Hospital of Wannan Medical College, Wuhu 241001 Anhui Province, PR China; b Anhui Province Key Laboratory of Non-coding RNA Basic and Clinical Transformation, No. 2, Zhe Shan Xi Road, Wuhu 241001 Anhui Province, PR China; c Inflammation and Immune Mediated Diseases Laboratory of Anhui Province (Anhui Medical University), Hefei 230032 Anhui Province, PR China; d Drug Research & Development Center, School of Pharmacy, Wannan Medical College, Wuhu 241002 Anhui Province, PR China; e Department of Orthopedics, The Second Hospital & Clinical Medical School, Lanzhou University, Lanzhou 730030 Gansu Province, PR China

**Keywords:** Osteosarcoma, hMSCs-derived exosomes, MIR17HG, miRNA-372-3p, BCL6, Tfh cell

## Abstract

**Background:**

Osteosarcoma (OS) is a life-threatening malignancy in children and adolescents, with limited treatment options for resistant or metastatic disease. Exosomes derived from hMSCs regulate tumor immunity by transporting molecules such as long non-coding RNA (lncRNA). The lncRNA MIR17HG is known to promote OS progression, yet its role in regulating T follicular helper (Tfh) cell differentiation in OS is unclear. This study is the first to systematically explore how MIR17HG influences Tfh cell differentiation, activation, and OS cell proliferation via the miR-372-3p/BCL6 signaling cascade.

**Methods:**

Exosomes were extracted from hMSCs using differential centrifugation. Characterization of hMSCs-derived exosomes was conducted by TEM, NTA and western blotting. The expression of MIR17HG, BCL6 and PD-1 was determined by qRT‒PCR or western blotting. The differentiation and activation of Tfh cells, as well as OS apoptosis, were assessed through flow cytometry. OS cell viability was determined by a CCK-8 assay. The effect of hMSCs-Exo-MIR17HG on tumors was assessed in an MG63-derived xenograft mouse model. The expression of Ki67 was examined via IHC. RNA immunoprecipitation was performed to validate the interaction between MIR17HG and miR-372-3p. A dual-luciferase reporter assay was performed to explore the correlation between miR-372-3p and BCL6.

**Results:**

The lncRNA MIR17HG was expressed in hMSCs-derived exosomes and upregulated by overexpression. hMSCs-Exo or hMSCs-Exo-MIR17HG promoted the differentiation and activation of Tfh cells, accompanied by increased PD-1 and BCL6 expression in CD4^+^ T cells, which enhanced OS cell proliferation. Furthermore, hMSCs-Exo-MIR17HG exerted a tumor-promoting effect in a CDX mouse model. Mechanistically, the effects driven by MIR17HG were abolished by miR-372-3p overexpression, and BCL6 was identified as a direct functional target of miR-372-3p.

**Conclusion:**

These findings demonstrate that exosomal MIR17HG derived from hMSCs drives the differentiation of follicular helper T cells and the progression of OS via the miR-372-3p/BCL6 axis.

## Highlights

• hMSCs-Exo-MIR17HG promote Tfh cell differentiation.
• hMSCs-Exo-MIR17HG promotes OS progression by enhancing Tfh cell differentiation.
• MIR17HG modulate Tfh cell differentiation and drive the progression of OS via the miR-372-3p/BCL6 signaling pathway.

## 1 Introduction

Osteosarcoma (OS) is one of the most common primary malignant tumors and is a severe threat to the lives and health of children and adolescents [1]. Despite significant progress made in recent decades, improving the overall survival of patients with osteosarcoma, particularly in cases of recurrence or metastasis, remains a major challenge [2]. Currently, the combination of surgical resection and chemotherapy has become the principal treatment modality for OS, but the prognosis of OS remains extremely unfavourable [3,4]. In addition, CAR-T cell therapy has demonstrated efficacy against OS in preclinical studies [4]. The pathogenesis of OS is complex and involves multiple factors. Abnormal changes in the immune system and tumor immunity are important causes of the onset or deterioration of OS [5]. For example, alterations in the quantity and function of immune cells, including dendritic cells, T lymphocytes, tumor-associated macrophages, tumor-associated neutrophils, and natural killer cells, may occur in patients with OS [6]. Follicular helper T cells (Tfh cells) represent a novel type of T cell that participates in regulating antibody production and immune memory formation and are closely associated with various malignant tumors [7]. For instance, a previous study suggested that Tfh cells derived from patients with non-small cell lung carcinoma triggered the differentiation of regulatory B cells and CD14^+^ human leukocyte antigen-DR-cells [8]. A greater percentage of Tfh cells in hepatocellular carcinoma is correlated with a worse prognosis [9]. Kang et al. confirmed that both the proportion and the activation level of Tfh cells in the peripheral blood of patients with OS were significantly elevated, which was related to poor prognosis [10]. These findings suggest that exploring the mechanism of Tfh cell differentiation may provide new insights and targets for OS therapy.

The extracellular vesicles secreted by mesenchymal stem cells (MSCs) belong to a class of small vesicular structures capable of delivering non-coding RNA, protein and lipids [11] and can participate in the modulation of the growth of numerous tumors. For example, Ge et al. reported that BMSC-derived exosomal LCP1 accelerates OS proliferation and metastasis through the JAK2/STAT3 pathway [12]. Similarly, BMSC-derived exosomal XIST facilitates the growth and metastasis of OS by downregulating the level of miR-655 [13]. Moreover, exosomes can affect tumor immunity, as exemplified by OS-derived exosomal PD-L1, which facilitates tumor growth by suppressing T cell activity [14].

Long non-coding RNAs (lncRNAs) are a type of non-coding RNA with a length of more than 200 nucleotides. LncRNAs are involved in modulating the growth, activation and differentiation of immune cells and affect the migration of immune cells and the release of cytokines [15]. Recently, the lncRNA IL21-AS1 was shown to be highly expressed in Tfh cells and to expedite the differentiation of Tfh cells through binding to hnRNPU [16]. The lncRNA MIR17HG, which is located at chromosome 13q31.3, participates in the regulation of cell proliferation, differentiation, and apoptosis [17]. Its aberrant expression is typically closely associated with the emergence of malignant tumors [18]. For example, the lncRNA MIR17HG promotes OS progression through the SP1/MIR17HG/miR-130a-3p/SP1 signaling cascades [19]. Therefore, we hypothesized that the lncRNA MIR17HG may regulate Tfh cell differentiation in OS, a possibility that has not yet been investigated.

miR-372-3p expression is reduced in OS tissues and cell lines. Additionally, *in vivo* overexpression of miR-372-3p suppresses tumor growth [20]. In addition, BCL6, recognized as a key transcription factor, enhances Tfh cell differentiation [21]. Evidence suggests that exosomes packaged with miR-101 inhibit OS tumor metastasis by targeting BCL6 [22]. Moreover, the ENCORI database (https://rnasysu.com/encori/) predicted that miR-372-3p has potential binding relationships with the lncRNA MIR17HG and BCL6.

In our study, we hypothesized that the lncRNA MIR17HG in extracellular vesicles derived from hMSCs promotes Tfh cell differentiation through the miR-372-3p/BCL6 axis, thereby promoting the progression of OS.

## 2 Methods

### 2.1 Cell culture

Human mesenchymal stem cells (hMSCs) were obtained from Wuhan Procell Biotechnology and cultivated in a specially formulated growth medium (Procell, China) supplemented with 10 % fetal bovine serum (Beyotime, C0235, China) and 1 % penicillin‒streptomycin (Beyotime, C0222). hMSCs were identified and purified according to marker expression confirmed in previous research (CD34^-^CD44^+^CD90^+^) by flow cytometry (FCM, BD, USA). CD4^+^ T cells were isolated from human PBMCs (SAILYBIO, China) with anti-human CD4 conjugated microbeads (Miltenyi Biotec, CA) and cultivated in RPMI 1640 medium (HyClone, SH30255.01, USA) supplemented with 10 % FBS (Beyotime, C0235), 1 % PS (Beyotime, C0222), 5 µg/mL anti-CD3 (ACROBiosystems, GMP-MC0323, China) and 5 µg/mL anti-CD28 (Invitrogen, 740009H, USA) after the purity was determined (>95 %) using flow cytometry (BD, USA). Human osteoblasts (MG63) were procured from YaJi Biotechnology (Shanghai, China) and grown in α-MEM (Gibco, 12571063, USA) supplemented with 10 % FBS, after which they were maintained at 37 °C and 5 % CO_2_.

### 2.2 Cell transfection

The overexpression vectors for MIR17HG (oe-MIR17HG), the empty vector (oe-NC), the overexpression vector for BCL6 (oe-BCL6), the miRNA-372-3p mimics, and the mimic negative control (NC mimics) were constructed and produced by GenePharma (Shanghai, China). First, hMSCs or CD4^+^ T cells were seeded into a six-well plate. In accordance with the relevant protocols, these cells were subsequently transfected with the designated plasmids using a Lipofectamine® 3000 kit (Invitrogen, USA). After 48 h, the transfected cells were collected for further analysis.

### 2.3 Extraction of exosomes from hMSCs

The cell culture supernatants containing exosomes from hMSCs transfected with oe-MIR17HG and oe-NC were collected when the cells reached 80 %-90 % confluence. Total exosome isolation reagent (Thermo Fisher Scientific, 44-783-60, USA) was subsequently used to isolate and purify the exosomes from the hMSCs in accordance with the instructions. Briefly, the harvested supernatants were spun at 2000×*g* for 30 mins to eliminate dead cells and cell debris. Afterward, 500 μL of total exosome isolation reagent was added to the samples, which were subsequently incubated at 4 °C overnight. Finally, the pellets with exosomes were retained and resuspended in 200 µl of PBS for identification or maintained at −80 °C after 60 mins at 10,000×*g*. The isolated exosomes were denoted as hMSCs-Exo, hMSCs-Exo-NC, and hMSCs-Exo-MIR17HG. All the centrifugations were performed at 4 °C.

### 2.4 Nanoparticle tracking analysis (NTA)

The exosome particle size was measured using a NanoSight LM10 HS microscope (NanoSight Ltd., UK) and analyzed with NanoSight NTA 2.3 software (Malvern, USA). Prior to analysis, the samples were diluted 1:100 in PBS to achieve optimal particle concentrations (10^6^–10^9^ particles/mL) and examined under a 488 nm laser source. All measurements were performed with six technical replicates per experimental group.

### 2.5 Transmission electron microscopy (TEM) observation

The obtained exosomes were diluted in PBS. Afterward, 5–10 μL of the exosome solution was carefully added onto a copper grid and incubated for 2 mins at ambient temperature. The extra liquid was meticulously absorbed with filter paper. Afterward, 10 μL of a 3 % aqueous phosphotungstic acid solution (pH 6.5) was gradually added dropwise to the copper grid, after which the exosomes were negatively stained for 5 mins at the same temperature. The copper grid was subsequently inspected under a transmission electron microscope (TEM, HITACHI, Japan). Finally, the surplus liquid was removed, and the grid was air-dried at room temperature.

### 2.6 Co-culture assay

CD4^+^ T cells were incubated with 50 μg/mL PBS or exosomes isolated from hMSCs (hMSCs-Exo, hMSCs-Exo-NC, and hMSCs-Exo-MIR17HG) for 4 days. Moreover, CD4^+^ T cells transfected with plasmids, namely, NC mimics, miRNA-372-3p mimics, miRNA-372-3p mimics + oe-NC, or miRNA-372-3p mimics + oe-BCL6, were prepared. These two groups of CD4^+^ T cells were then co-cultured with MG63 cells at a 3:1 ratio. During the co-culture, anti-CD3 (5 µg/mL, sourced from ACROBiosystems, GMP-MC0323) and anti-CD28 (5 µg/mL, from Invitrogen, 740009H) antibodies were added. After 72 h of incubation, the viability and apoptosis of the MG63 cells were evaluated.

### 2.7 Flow cytometric analysis

The hMSCs, CD4^+^ T cells, and MG63 cells were processed according to the designated treatment. First, the cells were harvested and then rinsed thoroughly with PBS. In accordance with the manufacturer's guidelines, the cells were subsequently incubated with the relevant antibodies at 4 °C. Afterward, all the cells were analyzed via a CytoFlex flow cytometer (manufactured by Beckman Coulter, USA). Finally, they were washed twice more with PBS. The hMSCs were incubated with PE-labeled anti-CD34 antibody (Proteintech, PE-65183, China), FITC-labeled anti-CD44 antibody (Abcam, ab30405, USA) or FITC-labeled anti-CD90 antibody (Abcam, ab124527). The purified CD4^+^ T cells were subjected to staining with only a FITC-labeled anti-CXCR5 antibody (Abcam, ab322305) to determine the proportion of CD4^+^CXCR5^+^Tfh cells within the CD4^+^T cell population. For CD4^+^CXCR5^+^Tfh cells, an additional staining step with a PE/Cy5.5-labeled anti-CD69 antibody (Abcam, ab272252) was performed to calculate the proportion of CD4^+^CXCR5^+^CD69^+^Tfh cells relative to total CD4^+^ T cells. To determine the proportion of CD4^+^CXCR5^+^CD69^+^Tfh cells among CD4^+^ T cells in mouse peripheral blood, the cells were first incubated with the relevant antibodies (anti-CD4-FITC (Invitrogen, 11-0049-42, USA), FITC-labeled anti-CXCR5 antibody (Invitrogen, 12-7185-82), and PE/Cy5.5-labeled anti-CD69 antibody (BioLegend, 104510, USA)). Among viable cells, the CD4^+^ cell population was gated. Within this CD4^+^T cell gate, the CXCR5^+^ cell population was initially gated, which represented the total Tfh cell population. Within the pregated CXCR5^+^ cell population, the expression of CD69 was subsequently analyzed. At this stage, CD69-positive cells correspond to CD4^+^CXCR5^+^CD69^+^Tfh cells. For the collected MG63 cells, 5–10 × 10^4^ cells were suspended in 195 μL of Annexin V-FITC binding buffer. Afterward, 5 μl of Annexin V-FITC (Beyotime, C1062S) and 10 μl of PI (Beyotime, ST511) were added. The mixture was incubated for 10–20 mins in the dark at room temperature and then transferred to flow cytometry tubes for detection.

### 2.8 Quantitative real-time polymerase chain reaction (qRT-PCR)

TRIzol reagent (Takara, Japan) was used to extract total RNA from hMSCs and CD4^+^ T cells that had been treated under various conditions. Next, the samples were quantified with a Nanodrop, and reverse transcription was performed using a QuantiTect Reverse Transcription Kit (QIAGEN, USA) following the manufacturer's protocol. The levels of the lncRNA MIR17HG and BCL6 were measured by qRT-PCR with a SYBR Premix Ex Taq II Kit (Takara, Japan) on a 7900HT system. β-actin or U6 served as the internal reference genes. The primer sequences are presented in Table 1, from the 5′ to 3′ terminus.

**Table 1:** The sequences of primers used for qRT-PCR.

Gene	Forward	Reverse
MIR17HG	GGCGTCCCGTCGTAGTAAAG	CATTGTGTCAGGAGTCAGTGTGTC
BCL6	CATGCAGAGATGTGCCTCCACA	TCAGAGAAGCGGCAGTCACACT
β-actin	TGGCACCACACCTTCTACAA	CCAGAGGCGTACAGGGATAG
U6	CTCGCTTCGGCAGCACAT	TTTGCGTGTCATCCTTGCG

### 2.9 Western blot

The cells from the hMSCs-Exo group, along with the CD4^+^ T cells that had been treated as described, were harvested, washed, and then lysed with RIPA lysis buffer (Beyotime). After quantification with either a BCA kit (Beyotime) or NanoDrop (Thermo Fisher Scientific, USA), 40 μg of protein was separated via SDS‒PAGE and transferred onto a polyvinylidene fluoride (PVDF) membrane (Millipore, IPVH00010, USA). Next, the membranes were blocked with 5 % skim milk powder for 2 h, after which they were incubated with different primary antibodies: rabbit anti-CD9 (Abcam, ab236630, 1:1000), rabbit anti-CD63 (Abcam, ab68418, 1 µg/mL) and rabbit anti-CD81 (Proteintech, 84644-1-RR, 1:10,000) for hMSCs-Exo group cells; rabbit anti-BCL6 (Abcam, ab272859, 1:500) and rabbit anti-PD-1 (Thermo Fisher Scientific, PA5-20351, 1 µg/mL) for the CD4^+^T cells; and rabbit anti-β-actin (Abcam, ab8227, 1:4000) for the above cells overnight at 4 °C. HRP-conjugated goat anti-rabbit IgG H&L antibody (Proteintech, SA00001-2, 1:5000) was subsequently incubated with the membranes at room temperature for 2 h. After being washed three times with TBST buffer, the membranes were developed with an enhanced chemiluminescence (ECL) kit (Solarbio, China) via a ChemiDoc imaging system (Bio-Rad, USA). Finally, the band intensity was quantified via ImageJ software (National Institutes of Health, USA), and the results were normalized to that of β-actin.

### 2.10 Cell counting kit-8 (CCK-8) assay

The viability of the MG63 cells co-cultured as per the instructions was evaluated using a CCK8 kit from Solarbio, China. First, a 10 % (v/v) CCK-8 solution was added to the wells in the culture medium. Afterward, the plate was placed in an incubator maintained at 37 °C for 2.5 h. Finally, the plate was inserted into a microplate reader (Bio-Rad, USA) to measure the optical density (OD) at 450 nm.

### 2.11 RNA immunoprecipitation (RIP)

The binding of MIR17HG to miR-372-3p was evaluated with a Magna RIP kit (Millipore, USA). Briefly, CD4^+^ T cells transfected with miR-NC mimics and miR-372-3p mimics were lysed and then incubated with rabbit anti-Ago2 polyclonal antibody (Bioss, bs-12450R)-coupled beads and anti-IgG antibody (Abcam, ab6715)-coupled beads at 4 °C. The level of MIR17HG was evaluated by qRT–PCR after the RNA was extracted from the samples using TRIzol.

### 2.12 Dual luciferase assay

The BCL6-WT and BCL6-MUT reporter vectors were procured from GenScript (Shanghai, China) and transfected into CD4^+^ T cells. After 48 h, the cells were plated in 96-well plates at a density of 10,000 cells per well and treated with NC mimics or miR-372-3p mimics 24 h after transfection. The luciferase intensity was subsequently measured by means of the Dual-Glo System (Promega, USA).

### 2.13 Xenograft assays

Male nude mice (5–6 weeks old, n = 18) were bought from Charles River (Beijing, China) and randomly grouped into 3 groups: the MG63 + PBS group, the MG63 + hMSCs-Exo-NC group and the MG63 + hMSCs-Exo-MIR17HG group for xenograft modeling, with 6 mice in each group. MG63 cells (2 × 10^6^ cells/100 μL) supplemented with PBS or 10 μg of hMSCs-derived exosomes (hMSCs-Exo-NC, hMSCs-Exo-MIR17HG) were subsequently injected subcutaneously into the axilla of the nude mice following the protocol. The tumor volume was measured every 7 days with a Vernier caliper (Mahr, Germany). Twenty-eight days later, the mice were euthanized. The tumors were dissected, weighed, and either kept at −80 °C or embedded in paraffin for further experiments. The animal experiments were approved by the Experimental Animal Welfare Committee of the First Affiliated Hospital of Wannan Medical College (approval no. WNMC-AWE-2025169).

### 2.14 Immunohistochemistry (IHC)

Paraffinized tumor sections were subjected to conventional dewaxing, and antigen retrieval was performed with microwaves in citrate-buffered solution (pH 6.0). The sections were blocked with 5 % bovine serum albumin (Sigma, USA) for 30 mins and then incubated with a rabbit anti-Ki-67 antibody (Abcam, ab16667, USA) for 90 mins and an HRP-labeled goat anti-rabbit IgG antibody (Abcam, ab6721, USA) at 4 °C for 20 mins in sequence. The sections were subsequently stained with DAPI solution (Solarbio, China), and the slides were subsequently photographed using a microscope (Leica Microsystems, Germany).

### 2.15 Bioinformatics analysis

The bioinformatics resource ENCORI (https://rnasysu.com/encori/), which systematically analyzes the interaction networks of miRNA-mRNA and miRNA-lncRNA, was used to predict the possible binding sites between lncRNA MIR17HG and miR-372-3p, as well as between miR-372-3p and BCL6.

### 2.16 Statistical analysis

For statistical processing, GraphPad Prism 10.1.2 (GraphPad Software, USA) and ImageJ (National Institutes of Health, USA) were used. The data are shown as the mean ± standard deviation (mean ± SD). To determine the statistical significance between two groups, Student’s *t* test was applied, and one-way analysis of variance (ANOVA) was used for comparisons among multiple groups. A p value less than 0.05 was considered to indicate statistical significance.

## 3 Results

### 3.1 Morphological characterization of hMSCs and exosomes

**Fig. 1:**
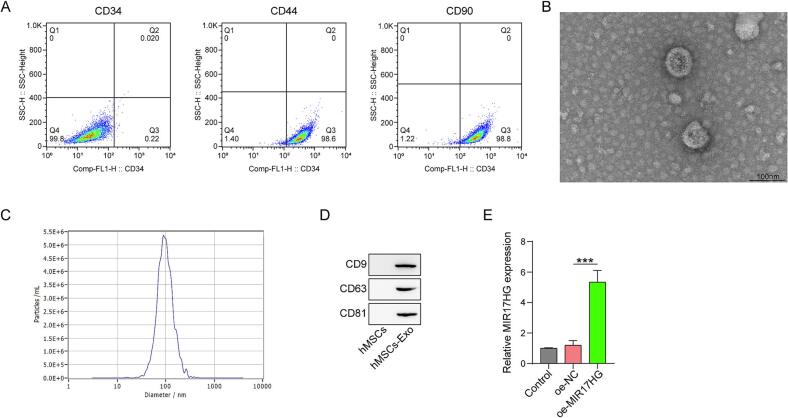
Morphological characterization of the hMSCs and hMSCs-derived exosomes. (A) The expression of CD34, CD44 and CD90 in hMSCs was determined by flow cytometry. (B)(C) Characterization of hMSCs-derived exosomes by TEM and NTA. (D) The expression of CD9, CD63 and CD81 in hMSCs-Exo were determined by flow cytometry. (E) The expression of the lncRNA MIR17HG in hMSCs was evaluated by qRT-PCR. Data were displayed as the mean ± SD. n = 3. oe-MIR17HG group compared with the oe-NC group, ***p < 0.001.

First, the hMSCs were characterized by flow cytometry. The results revealed that the hMSCs were positive for CD44 and CD90 expression, whereas the expression of CD34 was nearly undetectable. (Fig. 1A). Morphological observations of hMSCs-derived exosomes were subsequently performed using transmission electron microscopy (TEM). Electron micrographs revealed that the yielded exosomes were round or oval shaped (Fig. 1B). Moreover, the diameter of the isolated exosomes, characterized by nanoparticle tracking analysis (NTA), ranged from 60-100 nm, which is consistent with that of previously reported exosomes (Fig. 1C). In addition, CD9, CD63 and CD81, which were specifically expressed on the surface of the exosomes, were highly expressed in the obtained exosomes (Fig. 1D).

### 3.2 hMSCs-Exo-MIR17HG contributes to the differentiation of Tfh cells and further promotes the proliferation of OS cells

**Fig. 2:**
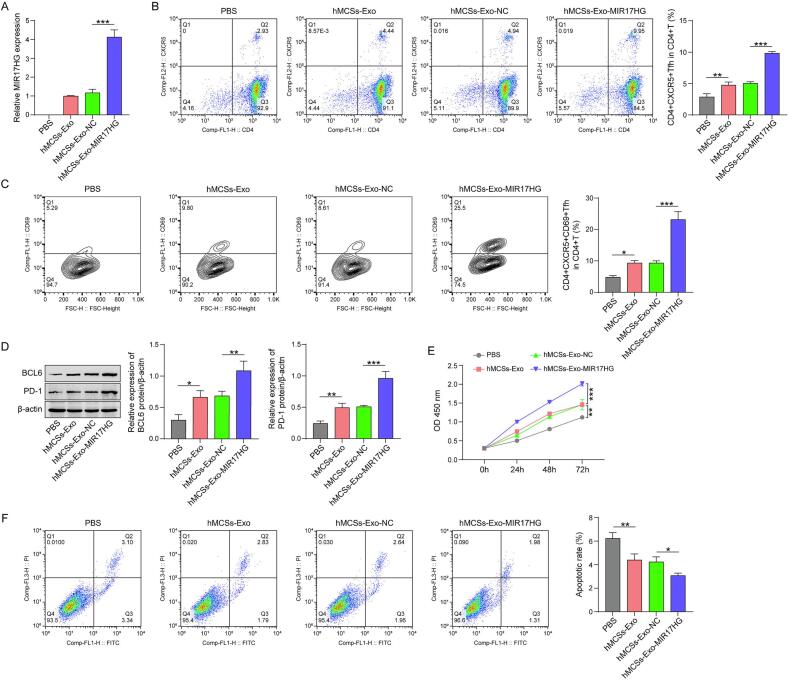
Hmscs-exo-mir17hg contributes to the differentiation of Tfh cells and further promotes the proliferation of OS cells. (A) The expression of MIR17HG in hMSCs-Exo was determined by qRT-PCR. (B)(C) Flow cytometry was used to evaluate the differentiation and activation of CD4^+^ T cells. (D) The expression of PD-1 and BCL6 in CD4^+^ T cells was assessed by western blotting. (E)(F) The viability and apoptosis of human OS cells were evaluated by a CCK-8 kit and flow cytometry, respectively. The data are displayed as the mean ± SD. n = 3, hMSCs-Exo group compared with the PBS group, hMSCs-Exo-MIR17HG group compared with the hMSCs-Exo-NC group, *p < 0.05, **p < 0.01, and ***p < 0.001.

Transfection of oe-MIR17HG led to a marked increase in MIR17HG expression in hMSCs (Fig. 1E). Consistent with these findings, the level of MIR17HG was notably increased in exosomes secreted by hMSCs (Fig. 2A). After the extracted exosomes were supplemented into CD4^+^ T cells, flow cytometry analysis revealed that upon treatment with hMSCs-Exos, the proportion of CD4^+^CXCR5^+^ Tfh cells among CD4^+^ T cells increased. Notably, hMSCs-Exo-MIR17HG further augmented this rise in percentage (Fig. 2B). Given that CD69 is recognized as a biomarker for the early activation of T cell subsets, we examined its expression levels in T cells. Upon the addition of hMSCs-Exo, the percentage of CD4^+^CXCR5^+^CD69^+^ Tfh cells in the CD4^+^T cell population clearly increased, especially when hMSCs-Exo-MIR17HG were applied (Fig. 2C). Moreover, treatment with hMSCs-Exo increased PD-1 and BCL6 expression in CD4^+^ T cells, as determined by western blotting, and was further enhanced by hMSCs-Exo-MIR17HG (Fig. 2D). Afterward, CD4^+^ T cells treated with exosomes were co-cultured with the human OS cell line MG63. The results of the CCK8 and flow cytometry assays indicated that CD4^+^ T cells treated with hMSCs-Exo-MIR17HG markedly increased the viability of MG63 cells and markedly inhibited apoptosis (Fig. 2E and F). These results indicate that treatment with hMSCs-Exo-MIR17HG promoted the differentiation and activation of Tfh cells and enhanced the viability of human OS cells.

### 3.3 hMSCs-Exo-MIR17HG promotes Tfh cell differentiation and further promotes OS progression

**Fig. 3:**
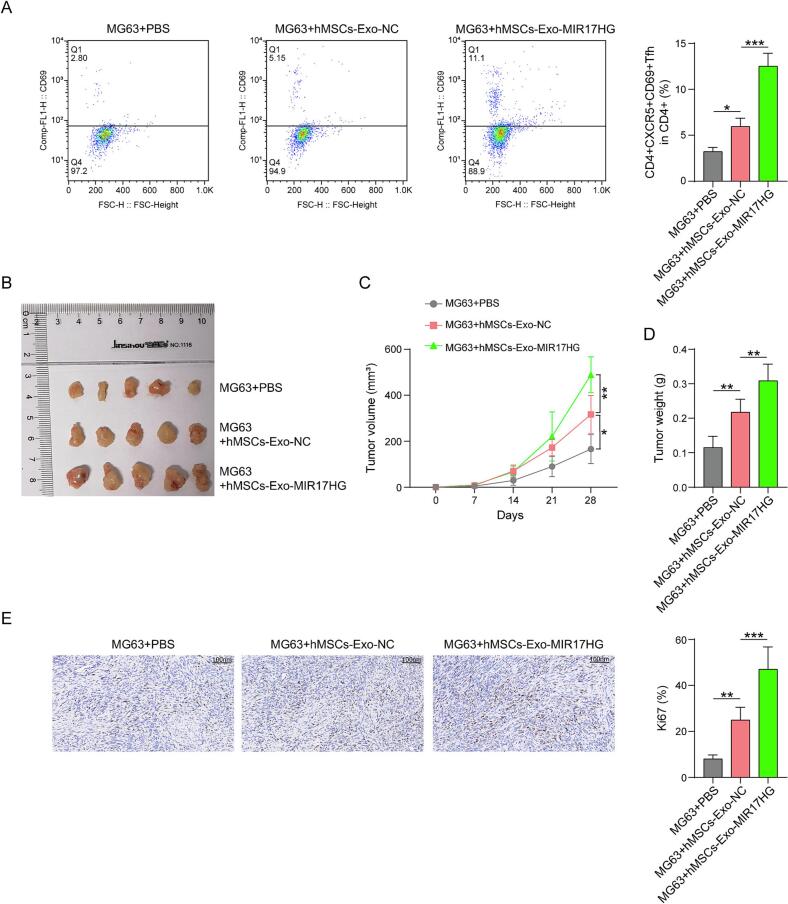
Hmscs-exo-mir17hg promotes Tfh cell differentiation and further promotes OS progression. (A) Flow cytometry was used to determine the proportion of CD4^+^CXCR5^+^CD69^+^ Tfh cells in peripheral blood. (B)(C)(D) The effect of hMSCs-Exo-MIR17HG on tumor growth and weight. (E) Immunohistochemistry was performed to determine the expression of Ki67. The data are displayed as the mean ± SD. n = 3, MG63 + hMSCs-Exo-NC group compared with the MG63 + PBS group, MG63 + hMSCs-Exo-MIR17HG group compared with the MG63 + hMSCs-Exo-NC group, *p < 0.05, **p < 0.01, and ***p < 0.001.

To further investigate the function of hMSCs-Exo-MIR17HG in OS progression, we set up a xenograft tumor model by subcutaneously injecting human OS cells into nude mice. Flow cytometry analysis revealed that, to some degree, the proportion of CD4^+^CXCR5^+^CD69^+^ Tfh cells in the peripheral blood of nude mice increased after the administration of oe-NC exosomes. A significant trend was observed for hMSCs-Exo-MIR17HG treatment (Fig. 3A). Furthermore, MG63 cells were subcutaneously injected into mice to investigate the regulatory role of hMSCs-Exo in tumor development. The tumor volume and weight moderately increased after 4 weeks of treatment. Furthermore, the hMSCs-Exo-MIR17HG group exhibited a significant tumor-promoting effect (Fig. 3B, C and D). The expression of Ki67 in tumors was increased following the use of hMSCs-Exo or hMSCs-Exo-MIR17HG, as detected by immunohistochemistry, and the latter was more significant (Fig. 3E). These results demonstrated *in vivo* that the hMSCs-Exo-MIR17HG promoted the differentiation of Tfh cells and subsequently promoted the progression of OS. However, the underlying mechanism remains unknown.

## 4 hMSCs-Exo-MIR17HG facilitates the differentiation of Tfh cells and promotes the proliferation of OS cells through binding to miRNA-372-3p

**Fig. 4:**
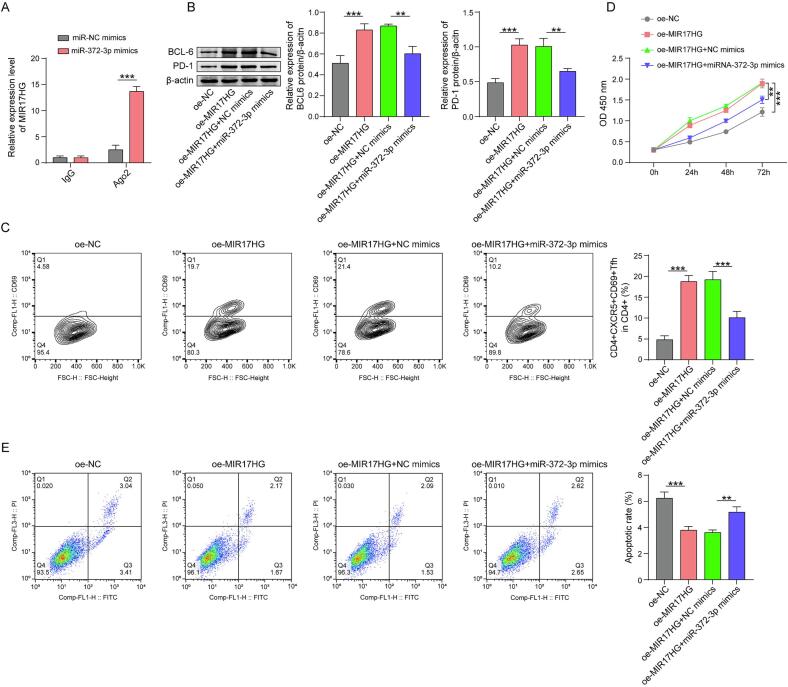
Hmscs-exo-mir17hg facilitates the differentiation of Tfh cells and promotes the proliferation of OS cells through binding to miRNA-372-3p. (A) RNA immunoprecipitation was performed to assess the interaction between MIR17HG and miR-372-3p. (B) Western blotting was performed to measure the expression of PD-1 and BCL6 in CD4^+^ T cells. (C) The differentiation of Tfh cells was determined by flow cytometry. (D)(E) The viability and apoptosis of human OS cells were determined by CCK-8 and flow cytometry, respectively. The data are displayed as the mean ± SD. n = 3, oe-MIR17HG group compared with the oe-NC group, oe-MIR17HG + miR-372-3p mimics group compared with the oe-MIR17HG + NC mimics group, **p < 0.01, ***p < 0.001.

According to the ENCORI database (https://rnasysu.com/encori/), miRNA-372-3p potentially binds to the lncRNA MIR17HG and BCL6. RNA immunoprecipitation assays verified that MIR17HG could indeed bind to miRNA-372-3p (Fig. 4A). MIR17HG overexpression increased PD-1 and BCL6 expression in CD4^+^ T cells. Transfection with miRNA-372-3p mimics significantly decreased the expression of these genes (Fig. 4B). Furthermore, oe-MIR17HG increased the proportion of CD4^+^CXCR5^+^CD69^+^ Tfh cells, whereas this increase was attenuated by supplementation with miRNA-372-3p mimics (Fig. 4C). In addition, the viability of hOS cells co-cultured with MIR17HG-overexpressing CD4^+^ T cells increased, whereas the apotosis of hOS cells decreased, and the addition of miR-372-3p mimics reversed these trends (Fig. 4D and E). These results demonstrated that MIR17HG combined with miRNA-372-3p may impede miRNA binding to target genes. Moreover, the overexpression of miRNA-372-3p mimics could counteract the ability of MIR17HG to promote Tfh cell differentiation and OS cell proliferation.

### 4.1 BCL6 is a direct target of miRNA-372-3p to promote Tfh differentiation and OS cell proliferation

**Fig. 5:**
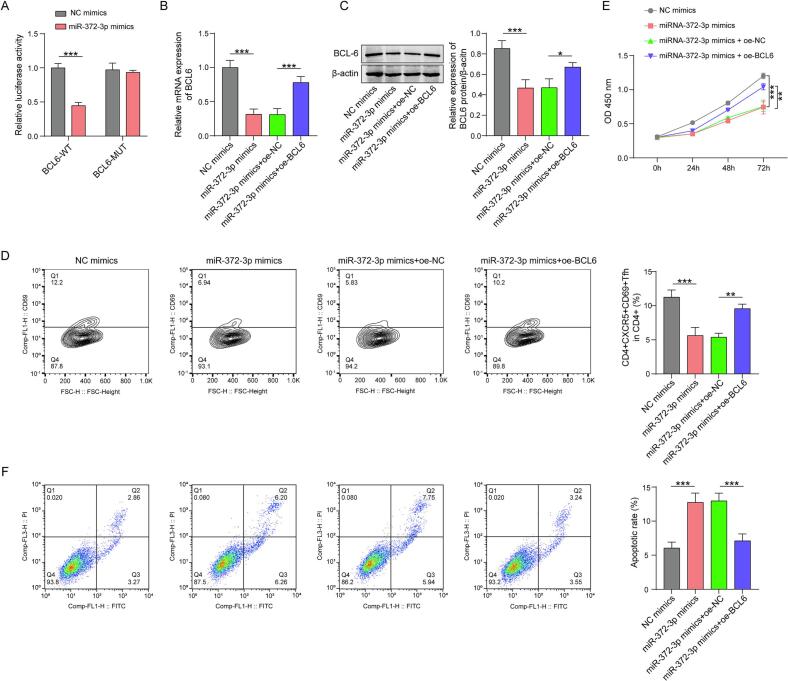
BCL6 is a direct target of miRNA-372-3p to promote Tfh differentiation and OS cell proliferation. (A) A dual-luciferase reporter assay was conducted to determine the association of miR-372-3p with BCL6. (B)(C) mRNA and protein expression was assessed by qRT-PCR and western blotting, respectively. (D) The differentiation of Tfh cells was determined by flow cytometry. (E)(F) The viability and apoptosis of human OS cells were determined by CCK-8 and flow cytometry, respectively. The data are displayed as the mean ± SD. n = 3, the miR-372-3p mimics group compared with the NC mimics, miR-372-3p mimics + oe-BCL6 group compared with the miR-372-3p mimics + oe-NC group, *p < 0.05, **p < 0.01, ***p < 0.001.

To confirm whether miRNA-372-3p binds to BCL6, we inserted the 3′UTR of BCL6 in either the wild-type (WT) or the mutant form before the reporter gene luciferase. It was observed that miRNA-372-3p mimics led to a decrease in luciferase activity in cells transfected with wild-type plasmids. However, this decrease was not significant in cells with mutant plasmids (Fig. 5A). When miRNA-372-3p mimics were transfected into cells, the levels of BCL6 mRNA and its related proteins in these cellular environments decreased significantly. Moreover, overexpression of BCL6 increased its own expression level (Fig. 5, Fig. 5). Compared with the NC mimics, the proportion of CD4^+^CXCR5^+^CD69^+^ Tfh within CD4^+^ T cells was decreased upon transfection of miRNA-372-3p mimics. Moreover, a reduction in cell viability and further enhanced apoptosis of human OS cells was observed. Nevertheless, the overexpression of BCL6 partially or even completely reversed these changes (Fig. 5D, E and F). These results strongly indicate that BCL6 is a direct target of miRNA-372-3p. The presence of miRNA-372-3p inhibited the differentiation and activation processes of Tfh cells.

## 5 Discussion

OS is among the most prevalent types of primary malignant tumors in children and adolescents [23]. Due to the complicated mechanism of OS pathogenesis, treatment currently relies mainly on surgical resection and chemotherapy [24]. Nevertheless, no highly effective method has been developed to treat chemotherapy-resistant, recurrent, and metastatic OS. Thus, gaining insights into the onset of OS and the development of novel clinical modalities is imperative. Exosomes are nanoscale extracellular vesicles that engage in cell–cell communication by transporting bioactive cargo molecules, leading to alterations in OS cell function and playing a significant role in the development of OS disease. For example, the BMSCs-derived exosomal lncRNA XIST can promote OS growth by interacting with miR-655 [13,25]. Furthermore, exosomal miR-21 can notably influence the plasticity of cancer cells, resulting in tumor progression, metastasis, angiogenesis and immune escape in OS [26]. In addition, BMSCs-Exo miR-21-5p increase OS cell proliferation and invasion by targeting PIK3R1 [27]. Thus, in-depth exploration of the role of exosomal lncRNAs or miRNAs in OS is highly important. The lncRNA MIR17HG has been shown to have diverse functions in many cancers, such as colorectal cancer, breast cancer and non-small cell lung cancer [18,[28], [29], [30]]. It also has a tumor-promoting function during the progression of OS, but the underlying mechanism remains unknown [19].

Here, we elucidate the mechanism through which hMSCs-derived exosomal MIR17HG promotes Tfh cell differentiation and OS progression. First, we characterized the morphological properties of the exosomes extracted from the hMSCs. The results revealed that these vesicles exhibited typical exosome characteristics, which were consistent with those reported in the literature. In addition, we found that hMSCs-derived exosomes expressed MIR17HG. Moreover, the transfection of MIR17HG in hMSCs significantly increased its expression in exosomes secreted by hMSCs. hMSCs-Exo-MIR17HG, as well as hMSCs-Exo, promoted Tfh formation and activation. Co-culture experiments revealed that hMSCs Exo-MIR17HG promoted OS cell proliferation by promoting the differentiation of Tfh cells. Similarly, the levels of PD-1, CXCR5 and BCL6 expression in CD4^+^ T cells were concurrently increased upon the addition of hMSCs-Exo or hMSCs-Exo-MIR17HG, which are regarded as biomarkers of Tfh cells [[31], [32], [33], [34]]. Furthermore, we investigated the impact of hMSCs-Exo-MIR17HG on the *in vivo* differentiation of CD4^+^ T cells. The percentage of CD4^+^CXCR5^+^CD69^+^ Tfh cells in the peripheral blood of nude mice clearly increased after the injection of hMSCs-Exo or hMSCs-Exo-MIR17HG. The intravenous administration of hMSCs-Exo-MIR17HG to MG63-derived xenograft mice promoted tumor progression. Collectively, our *in vitro* and *in vivo* data demonstrate that hMSCs-Exo-MIR17HG increase the differentiation of Tfh cells, thereby advancing the progression of OS tumors.

To elucidate how the lncRNA MIR17HG affects Tfh cell differentiation, RNA immunoprecipitation was performed. Strong interactions between MIR17HG and miR-372-3p were clearly detected. LncRNAs generally bind to miRNAs through a sponging mechanism, thus modulating the expression of downstream target genes. For example, the lncRNA CRNDE interacts with miR-181a-5p and subsequently increases the expression of TCF4 [35]. Indeed, hMSCs-Exo MIR17HG increased the cellular expression of PD-1 and BCL6 in CD4^+^ T cells. Further transfection of miR-372-3p counteracted the effects of MIR17HG on PD-1 and BCL6 expression, as well as on Tfh cell differentiation and activation. In addition, the viability of hOS cells co-cultured with oe-MIR17HG CD4^+^ T cells improved while the apoptosis decreased, and the addition of miR-372-3p mimics abolished these effects. Thus, the lncRNA MIR17HG affects cell differentiation and OS cell proliferation through miR-372-3p, which is consistent with the results predicted by bioinformatics. In addition, we explored the downstream target of miR-372-3p *in vitro*. The prediction that miR-372-3p would interact with BCL6 was verified by a dual-luciferase reporter assay. The mRNA and protein expression of BCL6 was concurrently reduced by miR-372-3p. The effects of miR-372-3p on Tfh cell differentiation and activation, as well as the viability and apoptosis of OS cells, could be counteracted by the transfection of oe-BCL6. In summary, BCL6 mediated the effects of miR-372-3p on cell differentiation and OS cell proliferation.

This study provides the first systematic investigation of how the exosomal lncRNA MIR17HG from hMSCs promotes Tfh cell differentiation and OS progression via the miR-372-3p/BCL6 axis. While a novel immunological mechanism was revealed, several limitations should be acknowledged. The dependency on cell lines and subcutaneous xenograft models, although convenient for tumor monitoring, fails to fully replicate the native bone microenvironment of OS, potentially limiting the translational relevance of our findings regarding tumor progression. Additionally, relatively small sample sizes in some experimental groups may have constrained statistical power, and despite the observed phenotypic alterations in Tfh cells, functional validation of their B cell helper capacity and antibody class-switching activity remains to be performed. Future investigations using orthotopic models, expanded clinical cohorts, and comprehensive Tfh functional assays will be crucial for further elucidating the role of this pathway in OS and assessing its therapeutic potential.

## 6 Conclusions

In conclusion, our study revealed that hMSCs-derived exosomal MIR17HG promotes Tfh cell differentiation via the miR-372-3p/BCL6 axis to increase OS growth, a finding corroborated by both *in vitro* and *in vivo* models.

## 7 Availability of data and materials

The datasets used or analyzed during the current study are available from the corresponding author upon reasonable request.

## Declaration of Generative AI and AI-assisted technologies in the writing process

The authors did not use any AI-assisted technology during the preparation of this work.

## CRediT authorship contribution statement

**Jin Qi:** Writing – review & editing, Writing – original draft, Supervision. **Gang Xue:** Writing – review & editing, Writing – original draft, Conceptualization. **Baomin Wu:** Writing – review & editing, Conceptualization. **Peng Zhu:** Writing – review & editing, Conceptualization. **Yapeng Wang:** Writing – review & editing, Visualization, Supervision, Project administration, Formal analysis, Data curation.

## Funding

This work was supported by the Major Natural Science Research Project of Anhui Provincial Department of Education (2024AH040249), the Talent Introduction Science Foundation of Yijishan Hospital, Wannan Medical College (YR20220214), the Inflammation and Immune Mediated Diseases Laboratory of Anhui Province (IMMDL202408), the Natural Science Foundation of Anhui Province (No. 2308085QH301), the Overseas Academic Visit Program founding for Anhui Provincial Young Backbone Teachers (No. JWFX2025032), the Key Natural Science Foundation of Anhui Higher Education Institutions (No.2022AH040171), and the 2022 Anhui Provincial Institute of Translational Medicine Research Project (2022zhyx-C48).

## Declaration of competing interest

The authors declare that they have no known competing financial interests or personal relationships that could have appeared to influence the work reported in this paper.
